# Multiscale Circuit Architecture Associated With Memory Dysfunction in Temporal Lobe Epilepsy

**DOI:** 10.1002/advs.76346

**Published:** 2026-07-09

**Authors:** Jiajie Mo, Qiwen Yuan, Pengfei Zhang, Lin Sang, Zhong Zheng, Baotian Zhao, Xiu Wang, Chao Zhang, Wenhan Hu, Xiaoqiu Shao, Jianguo Zhang, Ruijin Yang, Kai Zhang

**Affiliations:** ^1^ Department of Neurosurgery Beijing Tiantan Hospital Capital Medical University Beijing China; ^2^ McConnell Brain Imaging Centre Montreal Neurological Institute and Hospital McGill University Montreal Quebec Canada; ^3^ Department of Neurosurgery Ganzhou People's Hospital Ganzhou Jiangxi China; ^4^ Department of Neurosurgery Beijing Fengtai Hospital Beijing China; ^5^ Department of Neurosurgery Beijing Neurosurgical Institute Capital Medical University Beijing China; ^6^ Department of Neurology Beijing Tiantan Hospital Capital Medical University Beijing China

**Keywords:** cognitive systems neuroscience, mapping, memory dysfunction, normative modelling, temporal lobe epilepsy

## Abstract

Memory impairment is a major source of disability in temporal lobe epilepsy (TLE), yet how focal pathology relates to distributed circuit alterations underlying memory dysfunction remains unclear. We performed an integrative multiscale circuit‐level analysis in 250 patients with TLE to characterize memory dysfunction across focal structural damage, white matter disconnection, and distributed metabolic network organization, and to estimate individualized deviations in relation to memory performance. Auditory memory impairment was associated with damage to medial temporal structures centered on the hippocampus and parahippocampal cortex, as well as disruption of a key hippocampo‐cingulate white matter pathway. Memory deficits were linked to abnormal metabolic organization within a limbic‐centered network, with greater metabolic deviation associated with more severe global memory impairment. Spatial correspondence analyses showed that memory‐related hypometabolism aligns with serotonergic, GABAergic, and synaptic receptor distributions, with additional associations observed for inhibitory interneuron and mitochondrial signatures. Together, these findings delineate a multiscale circuit architecture underlying memory dysfunction. This framework provides a biologically grounded basis for understanding cognitive vulnerability and may inform individualized risk assessment in epilepsy surgery.

## Graphical abstract

A multiscale precision‐mapping framework reveals that memory impairment in temporal lobe epilepsy arises from the convergence of focal medial temporal pathology, strategic white matter disconnection, and limbic‐centered metabolic network dysfunction. Spatial correspondence with neurotransmitter and cellular systems further suggests a biologically structured vulnerability, offering a unified account of cognitive deficits and potential targets for individualized preservation.

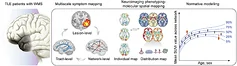

## 1 Introduction

Memory is a fundamental component of human cognition, enabling the encoding, consolidation and retrieval of experiences that support learning, decision‐making and daily function [1]. Disturbances of memory are therefore among the most disabling cognitive consequences of neurological disease. Temporal lobe epilepsy (TLE), the most common form of focal epilepsy, provides a uniquely informative human model for studying memory because its pathological processes frequently involve the hippocampal‐parahippocampal system and associated limbic circuits [2]. Although up to half of individuals with TLE exhibit clinically significant memory impairment, arising from both the disease and its surgical treatment, the neurobiological substrates underlying distinct memory deficits remain incompletely defined [3, 4].

Traditionally, memory dysfunction in TLE has been attributed to structural injury of the hippocampus [5]. Early neuroimaging studies supported a lateralized and domain‐specific model of memory organization, in which recollection deficits in unilateral TLE correspond to the functional specialization of the medial temporal lobe [6]. Task‐based paradigms showed that functional reorganization occurs predominantly within medial temporal structures [7], whereas widespread network involvement extending beyond the temporal lobe links to greater postoperative decline [8]. Extratemporal lobe regions, including the frontal, insular and cingulate cortices, have been shown to contribute to encoding and retrieval through their connectivity with hippocampal circuitry [9]. Yet, insights from these modalities have accumulated largely in parallel, each illuminating only one component of this multiscale system. A comprehensive framework for understanding the neuroanatomical basis of memory across multiple scales remains lacking, underscoring the need for an integrative, multiscale perspective to identify symptom‐critical regions and ultimately enable targeted cognitive preservation in TLE.

We address this gap by applying a multiscale precision‐mapping framework to a large cohort of patients with TLE. By integrating lesion‐, tract‐, and network‐level signatures with neurotransmitter/cellular correlates and normative modelling, we provide a unified account of how focal seizure foci and distributed circuit dysfunction jointly shape memory impairment in TLE. This multilevel approach reveals distinct anatomical and network fingerprints for each memory phenotype and identifies potential molecular systems involved in their selective vulnerability, providing mechanistic insights and a basis for individualized cognitive risk stratification in epilepsy surgery.

## 2 Materials and Methods

### 2.1 Participants

This study consecutively included patients diagnosed with TLE between May 2016 and December 2024 from Beijing Tiantan Hospital, and Beijing Fengtai Hospital. All patients underwent comprehensive evaluation, including long‐term video‐scalp electroencephalography (EEG) monitoring, brain magnetic resonance imaging (MRI), and [^18^F]fluorodeoxyglucose positron emission tomography ([^18^F]FDG PET) imaging, and intracranial stereoelectroencephalography (SEEG) when indicated [10, 11]. The epileptogenic zone (EZ) was localized through multidisciplinary case conferences integrating semiology, electrophysiology, structural imaging, metabolic imaging, and when available intracranial recordings. Surgical candidacy and resection strategy were established based on multidisciplinary consensus.

For the purposes of multiscale analysis, the TLE cohort was stratified into low‐grade epilepsy‐associated tumor/cerebral cavernous malformation (LEAT/CCM) and hippocampal sclerosis/malformations of cortical development (HS/MCD) subgroups based on the presence of intracranial space‐occupying effects on structural neuroimaging and confirmed histopathology. LEAT/CCM cases were used for voxel‐wise lesion delineation and tract‐level disconnection analyses, whereas HS/MCD cases were used for network‐level metabolic mapping, molecular association, and normative modelling. Eligible patients met the following criteria: (i) refractory epilepsy, defined as failure of adequate trials of at least two tolerated, appropriately chosen, and adequately dosed anti‐seizure medication (ASM) for a minimum of six months [12]; (ii) localization of the EZ in the temporal lobe based on detailed presurgical assessments; and (iii) underwent surgery with definitive postoperative histopathology. The exclusion criteria were as follows: (i) absence of surgical intervention; (ii) multilobar lesions; (iii) prior reoperation; (iv) neuromodulation; (v) hemispherectomy; (vi) dual pathology; (vii) encephalomalacia; (viii) history of traumatic brain injury; (ix) poor‐quality neuroimaging data, such as motion artifacts; (x) hydrocephalus.

The study followed the Strengthening the Reporting of Observational Studies in Epidemiology (STROBE) statement [13] and adhered to the principles of the Declaration of Helsinki. The Institutional Review Board of Beijing Tiantan Hospital (KY2020‐126‐01) approved anonymized data collection and sharing prior to the study's commencement. All patients or their guardians provided written informed consent to participate in the study.

### 2.2 Data Sources and Measurements

Demographics and clinical variables were extracted from medical records. All neuroimaging and neuropsychological assessments analyzed in this study were obtained during the presurgical evaluation period. Memory was assessed using the revised Chinese version of the Wechsler Memory Scale‐Fourth edition (WMS‐IV RC), including auditory memory index (AMI), visual memory index (VMI), visual working memory index (VWMI), immediate memory index (IMI), delayed memory index (DMI), and general memory index (GMI). All structural MRI scans were acquired on a 3T Siemens Verio scanner. The T1‐weighted magnetization prepared rapid gradient echo (MPRAGE) sequence parameters were: repetition time = 2300 ms, echo time = 2.53 ms, flip angle = 12°, slice thickness = 1 mm, no gap, voxel size = 1.0 × 1.0 × 1.0 mm^3^. Interictal [^18^F]FDG PET scans were performed under standardized resting conditions using the GE Discovery ST PET‐CT system (field of view = 300 mm, matrix = 192 × 192, slice thickness = 3.27 mm). Patients were instructed to rest quietly in a dimly lit room during the 40 min following the intravenous administration of [^18^F]FDG at a mean dose of 310 MBq/70 kg body weight. PET images were reconstructed using the ordered subset expectation maximization algorithm with 16 subsets and 6 iterations. All patients underwent [^18^F]FDG PET imaging under routine resting conditions within 6 months of presurgical evaluation, with no clinical seizures reported within 12 hours before or during the scan [14, 15].

### 2.3 Lesion‐Symptom Mapping (LSM)

To identify the focal anatomical substrates of memory impairment, we first performed LSM to localize brain regions where structural damage is associated with cognitive deficits. Lesions were manually delineated on structural MRI and normalized to the Montreal Neurological Institute (MNI152) standard space. Lesion segmentation on presurgical T1‐weighted images was independently performed by two senior neuroradiology‐trained raters, with discrepancies resolved by consensus or adjudicated by a third senior neuroradiologist to ensure reliability. Inter‐rater agreement was quantified using the Sørensen‐Dice similarity coefficient (Dice (A, B) = 2|A ∩ B| / (|A| + |B|)). The mean Dice coefficient was 0.84 ± 0.05, indicating high reproducibility of lesion delineation. Lesion burden was modeled as normalized lesion volume (lesion volume divided by total intracranial volume) and included as a covariate in all analyses.

We assessed the association between voxel‐wise lesion status (lesioned = 1, healthy = 0) and WMS‐IV indices using two complementary approaches: support vector regression (SVR) and the non‐parametric Brunner–‐Munzel (BM) test, enhancing robustness to model and distributional assumptions [16]. SVR yielded a voxel‐wise *z* map in which negative values indicate voxels where damage predicts poorer memory performance [17]. The non‐parametric BM test was applied to compare WMS‐IV scores between patients with and without a lesion at each voxel. To ensure sufficient statistical power, voxels lesioned in < 10% of patients were excluded from the analyses.

To assess the robustness of the LSM findings, we performed a three‐step validation procedure. First, voxel‐wise spatial correspondence between SVR *z* maps and BM *t* maps was quantified using Pearson's correlation, with ordinary least squares regression and 95% confidence intervals. Second, spatial autocorrelation was addressed using a spatially adjusted significance test based on 3D phase randomization. The BM *t* map was transformed into the Fourier domain, and its phase components were randomized while preserving the original amplitude spectrum, followed by inverse Fourier transformation to generate surrogate maps. Each surrogate BM map was correlated with the SVR *z* map to obtain one null correlation value. Repeating this procedure 5000 times yielded a null distribution against which the observed SVR‐BM correlation was evaluated to derive a spatially adjusted *p* value. Finally, a leave‐one‐out influence analysis was conducted by iteratively excluding each patient and recomputing SVR‐BM spatial correlations; the resulting change in similarity (Δ*ρ*) was used to quantify individual influence and its association with lesion volume.

### 2.4 Tract‐Symptom Mapping (TSM)

To determine whether the effects of focal lesions extend through white matter pathways, we next performed TSM to quantify lesion‐induced disconnection and its relationship with memory performance. TSM was performed using Brain Connectivity and Behaviour Toolkit (*BCBtoolkit*) to quantify both lesion‐tract overlap and lesion‐induced disconnectome maps [18]. Lesion masks in MNI space were first overlaid on a probabilistic Johns Hopkins University (JHU) white matter atlas derived from healthy controls [19]. For each tract, the lesion‐tract overlap proportion (overlapped volume divided by total tract volume) was computed for each patient and used as a continuous index of tract involvement for correlation and regression analyses with adjusted memory indices [20].

Lesion‐induced disconnectome maps were generated using diffusion‐weighted imaging data from the Human Connectome Project (HCP) dataset (n = 86, age = 29.7 ± 2.6 years, 60 female subjects), using whole‐brain tractography to track fibers passing through each lesion [19]. For each patient, the lesion mask was nonlinearly registered to each control's native diffusion space and used as a seed for tractography in *TrackVis* [21]. Resulting tractograms were converted into visitation maps, binarized, and warped back to Montreal Neurological Institute (MNI) space. Visitation maps were normalized and summed across controls to yield an individual disconnectome map, in which voxel values represent the probability (0%–100%) that fibers traversing that voxel would be disconnected by the lesion [22]. These disconnectome maps were entered into voxel‐wise multiple regression analyses implemented in Statistical non‐Parametric Mapping toolbox (*SnPM13*) [23], with memory indices as dependent variables and disconnection probability as the predictor of interest, yielding clusters of disconnection that were significantly associated with worse memory performance [24].

### 2.5 Network‐Symptom Mapping (NSM)

Given that memory dysfunction may reflect distributed network‐level alterations beyond focal lesions and tract disconnection, we next performed NSM to characterize metabolic network organization associated with memory impairment. First, PET images were rigidly coregistered to individual T1‐weighted images and spatially normalized to MNI space using deformation fields derived from structural normalization, with visual quality control. To reduce inter‐individual variability, PET images were normalized for injected dose and body weight and expressed as standardized uptake value ratio (SUVr) based on each subject's mean activity within the intracranial volume mask [25]. Partial volume effects were corrected using the voxel‐wise three‐compartment Müller‐Gärtner method [26]. Images were smoothed with a 6 mm full‐width at half‐maximum Gaussian kernel [27]. To account for site‐related variability, features were harmonized using *ComBat* with age and sex as covariates [28].

We performed a voxel‐wise general linear model (GLM) for PET intensity in healthy controls using age and sex as covariates to derive the normative model. For each patient, voxel‐wise metabolic *w*‐scores were computed as (observed – expected)/residual standard deviation (RSD), where expected values and RSD were derived from the normative model [29, 30]. The *w*‐maps were thresholded at *w* ≤ −1.96 SD below the normative mean. Individual metabolic network maps were then generated by linking each patient's binarized hypometabolic map to a normative functional connectome. Specifically, the mean time series within each metabolic map was extracted and correlated with voxel‐wise blood‐oxygenation‐level‐dependent (BOLD) signals using resting‐state fMRI data from 493 healthy individuals (age = 29.2 ± 3.4 years, 202 female subjects) in the HCP dataset [31]. Correlation coefficients were converted to a normal distribution using Fisher's *r*‐to‐*z* transform and entered into a single‐group voxel‐wise *t*‐test to generate patient‐specific metabolic network *t*‐maps [32, 33].

Finally, these metabolic network maps were entered into voxel‐wise multiple regression analyses implemented in SnPM13, with memory indices as predictors of interest and demographic variables included as nuisance regressors. Voxel‐wise NSM results were thresholded for statistical significance and summarized at the network level by intersecting significant maps with the Yeo 7‐network parcellation. For each memory index, the proportion of suprathreshold voxels within each network was calculated and used as an index of network‐level metabolic involvement, which was subsequently related to WMS performance.

### 2.6 Normative Modelling and Individual Deviations

To further quantify individual‐level deviations from normative brain organization, we applied normative modelling to estimate subject‐specific abnormalities in network‐level metabolism. Normative modelling provides a framework for quantifying individual deviations from population‐level reference trajectories, enabling the characterization of heterogeneous brain abnormalities beyond group‐level contrasts [34]. For each Yeo 7‐functional network showing significant effects, we performed normative modelling of PET metabolism in healthy controls using Bayesian linear regression with B‐spline basis functions, adjusting for age and sex [35]. The resulting models were then applied to individuals with HS/MCD pathology to estimate normative PET metabolic values for each region. Individual deviations were quantified using normative probability maps, computed as *z*‐scores reflecting the difference between observed and predicted metabolic values normalized by model uncertainty [36]. All normative models were implemented using the Predictive Clinical Neuroscience toolkit (*PCN*) [37], and linear regression analyses were performed to assess associations between network‐level deviation scores and WMS indices.

### 2.7 Spatial Correlation of Metabolic Abnormalities With Neurotransmitter and Cellular Markers

To link network‐level metabolic abnormalities to underlying neurobiological organization, we performed spatial correspondence analyses using the *JuSpace* toolbox [38]. The unthresholded NSM *t*‐statistic map for GMI, capturing voxel‐wise associations between PET metabolism and memory performance in the HS/MCD subgroup, served as the imaging input for subsequent analyses. This map was spatially correlated with normative molecular templates indexing major neurotransmitter receptors/transporters systems as well as transcriptomic‐derived cellular/mitochondrial signatures. Spearman rank correlations were computed between regional NSM effects and each molecular map. To mitigate confounding by regional tissue composition and spatial autocorrelation, all analyses were performed as partial correlations controlling for gray‐matter probability, and statistical significance was assessed using orthogonal permutation testing (Figure 1A).

**FIGURE 1:**
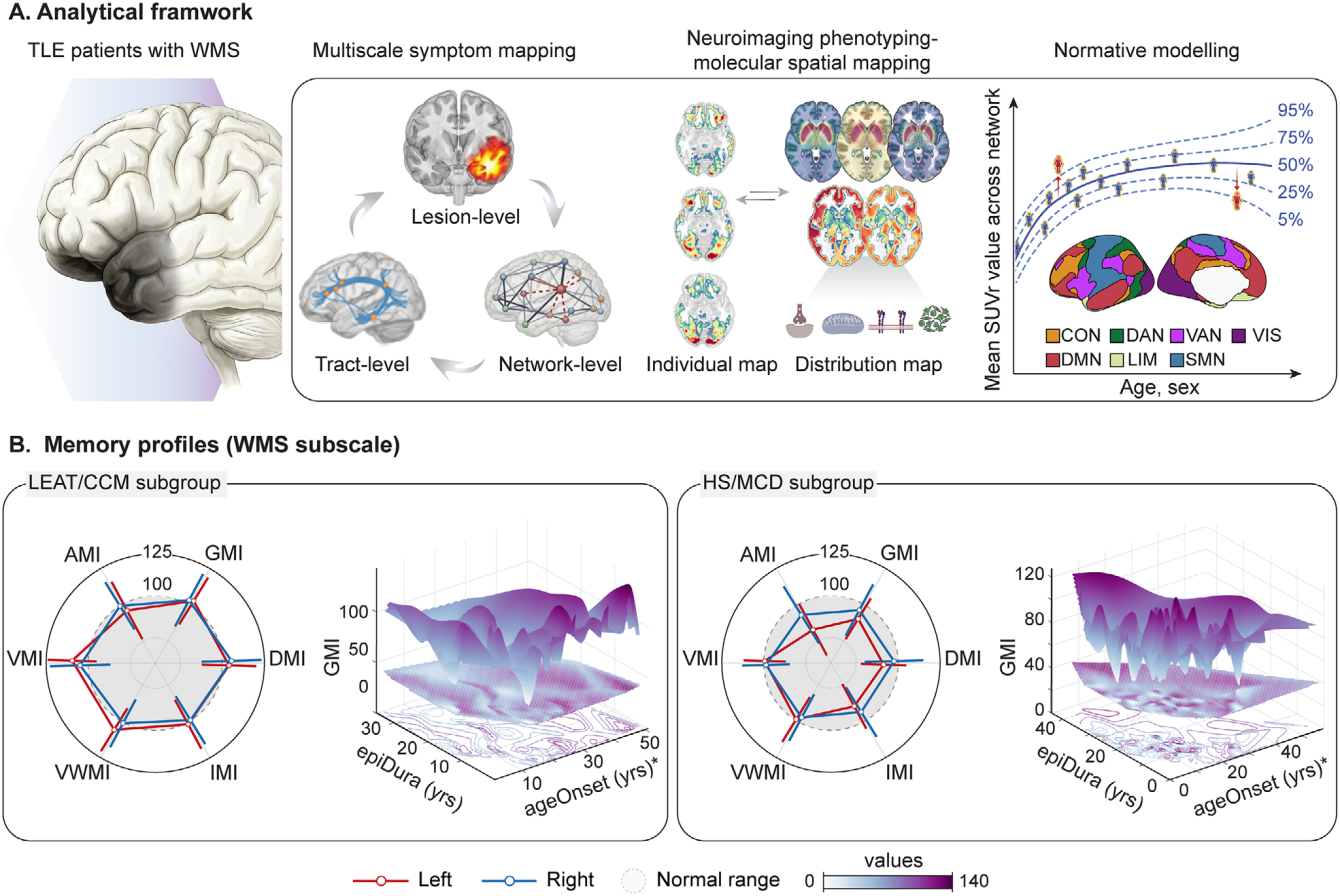
Analytical framework and memory profiles in temporal lobe epilepsy (TLE). (A) Analytical framework. The study integrated multiscale symptom mapping, including lesion‐, tract‐, and network‐level analyses, together with neuroimaging phenotyping, molecular spatial mapping, and normative modelling to characterize memory dysfunction in TLE. Individual metabolic maps were derived from normative modelling and subsequently linked to functional network organization and molecular templates to enable cross‐scale interpretation; (B) Memory profiles of LEAT/CCM and HS/MCD subgroups. Radar plots illustrated group‐level Wechsler Memory Scale–Fourth Edition (WMS‐IV) index scores across subscales for left‐sided (red) and right‐sided (blue) TLE, with the shaded region indicating the normative range (mean = 100). Three‐dimensional surface plots depicted the distributions and relationship between global memory performance (GMI) and clinical variables, including age at seizure onset and epilepsy duration, illustrating smooth trends derived from fitted models. Pearson's correlation coefficient (*r)* was used for correlation statistical analysis. Asterisks indicated statistically significant group differences (^*^: *P* < 0.05). Abbreviations: WMS‐IV, Wechsler Memory Scale–Fourth Edition; TLE, temporal lobe epilepsy; LEAT, low‐grade epilepsy‐associated tumor; CCM, cerebral cavernous malformation; HS, hippocampal sclerosis; MCD, malformations of cortical development; AMI, auditory memory index; VMI, visual memory index; VWMI, visual working memory index; IMI, immediate memory index; DMI, delayed memory index; GMI, general memory index; ageOnset, age at seizure onset; epiDura, epilepsy duration; CON, control network; DAN, dorsal attention network; DMN, default mode network; LIM, limbic network; VAN, ventral attention network; SMN, somatomotor network; VIS, visual network.

### 2.8 Statistical Analysis

Continuous variables were assessed for normality and homogeneity of variance and analyzed using parametric or nonparametric tests as appropriate. Two‐group comparisons used independent‐samples *t* tests (with Welch's correction) or Mann–Whitney *U* tests. Comparisons involving more than two groups were performed using one‐way analysis of variance (ANOVA), Welch's ANOVA when variances were unequal, or the Kruskal–Wallis H test for non‐normally distributed data. kal–Wallis *H* test. Categorical variables were compared using Pearson's *χ^2^* test or Fisher's exact test, as appropriate.

All analyses were conducted within a brain‐wide association framework [39]. To control for multiple testing across the analytical pipeline, voxel‐wise neuroimaging analyses (lesion‐, tract‐, and network‐symptom mapping) were performed using permutation‐based inference (5000 permutations). For all statistical models, WMS‐IV indices were adjusted for relevant demographic and clinical covariates, including sex, age at seizure onset, epilepsy duration, years of education, lesion volume, ASM burden, SEEG status, follow‐up duration, and handedness. All tests were two‐sided, with statistical significance set at false discovery rate (FDR)‐corrected *P* < 0.05.

## 3 Results

### 3.1 Demographic and Clinical Characteristics

The study inclusion process was summarized in Figure S1. Of 581 patients with TLE evaluated for epilepsy surgery, 84 with LEAT/CCM and 166 with HS/MCD pathology met inclusion criteria. Fifty‐two healthy controls (HCs) were recruited, of whom 33 were demographically matched for comparison. Demographic characteristics were comparable across groups. Age did not differ among the three groups (25.0 [13.0] vs 28.0 [10.8] vs 33.0 [23.0] years; *F* = 1.14, *P* = 0.321), nor did sex distribution (female: 42.9% vs 40.4% vs 57.6%; *χ^2^* = 3.33, *P* = 0.189). Compared with HS/MCD subgroup, LEAT/CCM subgroup showed a later seizure onset and shorter disease duration (18.0 [12.3] vs 13.0 [11.0] years, *Z* = 3.92, *P* < 0.001; 6.0 [11.8] vs 13.0 [7.0] years, *Z* = ‐4.82, *P* < 0.001), while years of education were slightly higher but did not reach statistical significance (15.0 [4.8] vs 12.0 [7.0] years, *Z* = 1.88, *P* = 0.062). The laterality of the epileptogenic zone was similar between patient groups (left side: 48.8% vs 56.6%; *χ^2^* = 1.37, *P* = 0.241), whereas SEEG implantation was markedly more frequent in the HS/MCD subgroup (4.8% vs 48.8%; *χ^2^* = 48.19, *P* < 0.001).

Memory assessment revealed significantly better memory performance in LEAT/CCM subgroup compared with HS/MCD subgroup across all WHS‐IV indices, including AMI (*t* = 4.13, *pP* < 0.001), VMI (*Z* = 3.83, *P* < 0.001) VWMI (*Z* = 2.47, *P* = 0.015), IMI (*t* = 4.16, *P* < 0.001), DMI (*t* = 4.92, *P* < 0.001), and GMI (*t* = 4.63, *P* < 0.001). Within the HS/MCD subgroup, age at seizure onset, years of education, sex distribution, and SEEG implantation were not statistically different. However, the left‐sided group showed a longer epilepsy duration (15.48 ± 8.62 vs 12.22 ± 7.78 years; *t* = ‐2.52, *P* = 0.013). On the WMS‐IV, right‐sided cases outperformed left‐sided cases on AMI (94.19 ± 19.39 vs 82.15 ± 17.55; *t* = 4.19, *P* < 0.001), IMI (94.61 ± 17.21 vs 89.03 ± 15.04; *t* = 2.22, *P* = 0.027), DMI (98.15 ± 16.75 vs 90.96 ± 14.01; *t* = 3.01, *P* = 0.003), and GMI (96.18 ± 16.87 vs 90.52 ± 14.56; *t* = 2.32, *P* = 0.022), whereas VMI and VWMI did not differ between sides (Table 1). In addition, age at seizure onset was modestly but significantly associated with GMI in both LEAT/CCM (*r* = 0.22, *P* = 0.049) and HS/MCD subgroup (*r* = 0.18, *P* = 0.022), whereas epilepsy duration showed no significant association (Figure 1B and Figure S2).

**TABLE 1:** Demographic and clinical characteristics.

Variables	LEAT/CCM subgroup (*n* = 84)	HS/MCD subgroup (*n* = 166)	HCs (*n* = 33)	Statistical analysis
**Clinical characteristics**				
Age (yrs)	25.0 (22.0, 35.0)	28.0 (22.3, 33.0)	33.0 (19.0, 42.0)	*F* = 1.14 *P* = 0.321
Sex (Female, %)	36 (42.9%)	67 (40.4%)	19 (57.6%)	*χ^2^* = 3.33 *P* = 0.189
Side of EZ (left, %)	41 (48.8%)	94 (56.6%)	NA	*χ^2^* = 1.37 *P* = 0.241
Age at seizure onset (yrs)	18.0 (13.0, 25.3)	13.0 (8.0, 19.0)	NA	*Z* = 3.92 *P* < 0.001
Epilepsy duration (yrs)	6.0 (2.5, 14.3)	13.0 (8.0, 19.8)	NA	*Z* = ‐4.82 *P* < 0.001
Seizure frequency (n, %)	Daily (13, 15.5%) Weekly (30, 35.7%) Monthly (25, 29.8%) Yearly (16, 19.0%)	Daily (24, 14.5%) Weekly (54, 32.5%) Monthly (81, 48.8%) Yearly (7, 4.2%)	NA	*χ^2^* = 18.30 *P* < 0.001
ASM burden [1]	1.3 (0.7, 1.9)	1.4 (0.9, 2.2)	NA	*Z* = ‐2.16 *P* = 0.062
Handedness (n, %)	Left (10, 11.9%) Right (68, 81.0%) Ambidextrous (6, 7.1%)	Left (12, 7.2%) Right (144, 86.7%) Ambidextrous (10, 6.0%)	NA	*χ^2^* = 1.72 *P* = 0.08
Education (yrs)	15.0 (11.3, 16.0)	12.0 (9.0, 16.0)	NA	*Z* = 1.88 *P* = 0.062
SEEG (Yes, %)	4 (4.8%)	81 (48.8%)	NA	*χ^2^* = 48.19 *p* < 0.001
Pathology (n, %)	CCM (13, 15.5%) LEAT (71, 84.5%)	MCD^2^ (44, 26.5%) HS (122, 73.5%)	NA	NA
Seizure freedom (n, %)	47/57 (82.5%)	99/140 (70.7%)	NA	*χ^2^* = 2.91 *P* = 0.088
**Wechsler memory scale‐Fourth Edition (WMS‐IV)**				
AMI	97.8 ± 18.0	87.4 ± 19.3	NA	*t* = 4.13 *P* < 0.001
VMI	106.0 (96.0, 117.0)	99.0 (89.0, 106.5)	NA	*Z* = 3.83 *P* < 0.001
VWMI	105.0 (90.0, 115.0)	100.0 (85.0, 110.0)	NA	*Z* = 2.47 *P* = 0.015
IMI	100.5 ± 16.5	91.5 ± 16.2	NA	*t* = 4.16 *P* < 0.001
DMI	104.7 ± 17.0	94.1 ± 15.6	NA	*t* = 4.92 *P* < 0.001
GMI	103.0 ± 16.9	93.0 ± 15.8	NA	*t* = 4.63 *P* < 0.001

Abbreviations: TLE, temporal lobe epilepsy; HCs, healthy controls; EZ, epileptogenic zone; SEEG, stereoelectroencephalography; CCM, cerebral cavernous malformation; LEAT, low‐grade epilepsy‐associated tumor; HS, hippocampal sclerosis; AMI, auditory memory index; VMI, visual memory index; VWMI, visual working memory index; IMI, immediate memory index; DMI, delayed memory index; GMI, general memory index; NA, not available.

Continuous variables were presented as mean ± standard deviation, or median (interquartile range), and categorical variables as counts (percentage). Group comparisons: *F*, one‐way ANOVA; *t*, independent samples *t* test; *Z*, Mann–Whitney *U* test (normal approximation); *χ^2^*, Pearson's chi‐square test. Normality was assessed with the Shapiro–Wilk test and homogeneity of variances with Levene's test; when assumptions were violated, Welch's corrections or non‐parametric tests were used.

[1] Preoperative anti‐seizure medication (ASM) regimens were re‐extracted and standardized using the World Health Organization (WHO) defined daily dose (DDD) framework to quantify medication burden.
[2] Malformations of cortical development (MCD) included focal cortical dysplasia (FCD) and mild MCD (mMCD).

### 3.2 Auditory Memory Deficits Linked to Medial Temporal Lesions Identified by LSM

Group‐level lesion overlap was shown in Figure 2A. In the LEAT/CCM subgroup, the AMI showed the most consistent deviation from normative performance across both left‐ and right‐sided cases, whereas other WMS‐IV indices remained largely within the normative range. We therefore focused on AMI to identify the neural substrates associated with auditory memory variability in LEAT/CCM subgroup. At the voxel level, both multivariate SVR‐LSM and non‐parametric BM analyses consistently implicated medial temporal lobe structures in auditory memory impairment, with the strongest effects observed for left‐sided lesions. In the main SVR results, significant clusters (permutation‐corrected, *P*_FDR_ < 0.05) were localized to the left hippocampus (*z* = −2.75, MNI coordinates: x = −26, y = −22, z = −15), left temporal pole (TP, *z* = −2.70, MNI coordinates: x = −44, y = 9, z = −39), and right parahippocampal gyrus (PHG, *z* = −2.69, MNI coordinates: x = 30, y = −26, z = −23). Complementary BM tests yielded convergent findings in overlapping regions (Figure 2B). Quantification of regional involvement demonstrated that auditory memory deficits were most strongly linked to lesions affecting medial temporal structures (Figure 2C and Table S1).

**FIGURE 2:**
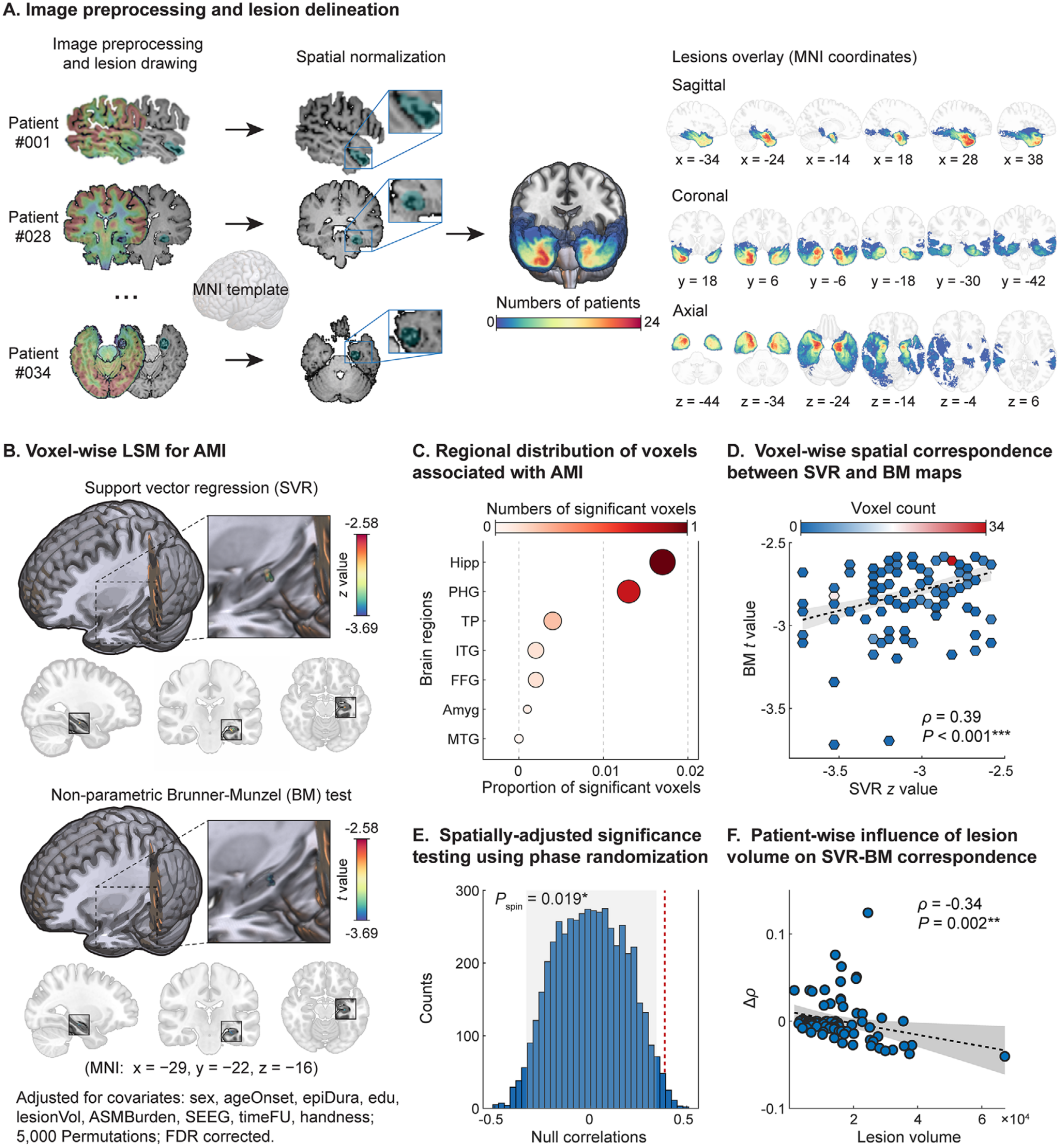
Lesion‐symptom mapping (LSM) identified medial temporal regions associated with memory impairment. (A) Image preprocessing and lesion delineation. Individual lesion masks from patients (*n* = 84) were manually delineated in native space and spatially normalized to MNI space. Group‐level lesion overlap maps were shown in sagittal, coronal, and axial views, with color intensity indicating the number of patients with lesions at each voxel; (B) Voxel‐wise LSM for auditory memory index (AMI). Multivariate support vector regression (SVR) and non‐parametric Brunner‐Munzel (BM) analyses were performed to identify brain regions associated with AMI, adjusting for demographic and clinical covariates. Statistically significant effects were shown, with analyses restricted to voxels lesioned in at least 10% of patients; (C) Regional distribution of LSM effects. Bubble plot summarized the proportion of significant voxels within temporal lobe regions, highlighting preferential involvement of medial temporal structures, including the hippocampus and parahippocampal gyrus; (D) Spatial correspondence between SVR and BM maps. Hexagonal‐bin scatter plots depicted voxel‐wise correspondence between SVR‐derived *z* maps and BM‐derived *t* maps. Color intensity reflected voxel density, and the regression line indicates the fitted linear relationship; (E) Spatially adjusted significance testing. A permutation‐based null distribution was generated using three‐dimensional phase randomization to account for spatial autocorrelation. The observed spatial correlation between SVR and BM maps (red dotted line) exceeded the null distribution, indicating robust convergence between methods; (F) Subject‐wise influence on map correspondence. A leave‐one‐out analysis was performed to assess the influence of individual lesions on spatial correspondence between SVR and BM maps. Changes in correlation (Δ*ρ*) were plotted against lesion volume, demonstrating that larger lesions exert greater influence, but no single patient dominated the group‐level pattern. Spearman's rank correlation test (*ρ*) test was used for statistical analysis (^*^: *P* < 0.05; ^**^: *P* < 0.01; ^***^: *P* < 0.001). Abbreviations: MNI, Montreal Neurological Institute; WMS‐IV, Wechsler Memory Scale–Fourth Edition; AMI, auditory memory index; Hipp, hippocampus; PHG, parahippocampal gyrus; TP, temporal pole; ITG, inferior temporal gyrus; FFG, fusiform gyrus; Amyg, amygdala; MTG, middle temporal gyrus.

Direct comparison of SVR and BM statistical maps showed voxel‐wise spatial correspondence (*r* = 0.39, *P* < 0.001; Figure 2D). This association remained significant after controlling for spatial autocorrelation using phase‐randomization testing (*r* = 0.39, *P_perm_* = 0.019), indicating that convergence between methods was not driven by lesion smoothness (Figure 2E). Leave‐one‐out influence analysis demonstrated that although larger lesions contributed more strongly to overall map similarity (*ρ* = −0.34, *P* = 0.002), removal of any single patient produced minimal change in SVR‐BM correspondence (Δ*ρ* < 0.1; Figure 2F).

Together, these findings indicated that auditory memory impairment in LEAT/CCM subgroup is preferentially associated with disruption of a medial temporal network centered on the hippocampus and PHG, with maximal vulnerability in the left hemisphere.

### 3.3 Cingulum‐Hippocampal Tract as a Critical WM Substrate of Memory Impairment

To determine whether temporal lesions impair memory via disruption of specific white matter pathways, TSM analysis comprised two complementary approaches: (1) assessment of associations between lesion–tract overlap and WMS indices, and (2) voxel‐wise statistical mapping based on individual disconnectome profiles (Figure 3A). Lesion‐tract overlap analysis demonstrated an asymmetric distribution of tract involvement, with the largest burdens in left ventral temporal association pathways and limbic fibers, including the basal forebrain‐amygdaloid fasciculus (SAF_ITG_MTG), cingulum‐hippocampal tract (CGH), uncinate fasciculus (UNC) and inferior longitudinal fasciculus (ILF), whereas homologous right‐sided tracts and corpus callosum showed smaller effects (Figure 3B). Across all tracts, only the left CGH demonstrated a statistically significant association with auditory memory impairment (*r* = −0.69, *P*_FDR_ < 0.001), whereas other heavily involved pathways, including SAF_ITG_MTG, showed no significant relationship with performance (*r* = −0.03, *P*_FDR_ = 0.860; Figure 3C and Figure S3). This effect was not simply driven by tract size, as the lesion‐tract overlap proportion showed only a modest and non‐significant relationship with tract volume across all tracts (*r* = −0.37, *P* = 0.113; Figure 3D).

**FIGURE 3:**
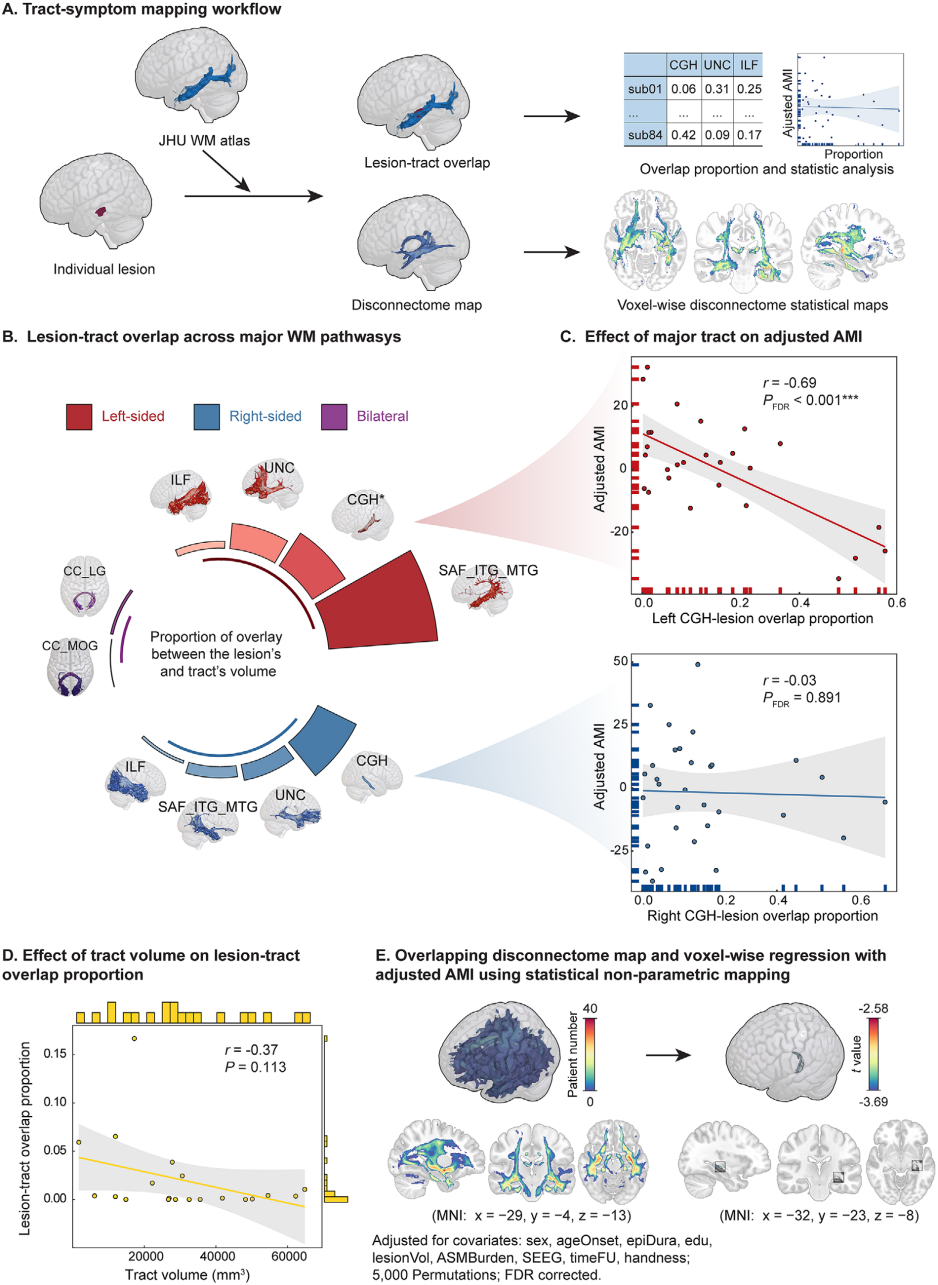
Tract‐symptom mapping (TSM) identified white matter pathways associated with memory impairment in TLE. (A) Analytical workflow of TSM. Individual lesion masks were intersected with a probabilistic JHU white matter atlas to estimate lesion‐tract overlap and derive tract‐level disconnection measures. For each patient, overlap proportions were computed and related to memory performance. In parallel, voxel‐wise disconnectome maps were generated to assess spatial patterns of disconnection associated with memory deficits; (B) Lesion‐tract overlap across major white matter pathways. Radial plots summarized the proportion of lesion‐tract overlap across tracts, grouped by laterality (left, right, and bilateral). Representative tract anatomy was shown for visualization, highlighting preferential involvement of limbic and temporo‐frontal pathways; (C) Association between tract involvement and memory performance. Scatter plots illustrated the relationship between lesion overlap within the cingulum‐hippocampal tract (CGH) and adjusted auditory memory index (AMI). A significant negative association was observed for left‐sided CGH involvement, whereas no association was detected for the right hemisphere; (D) Effect of tract volume on overlap proportion. A scatter plot showed the relationship between tract volume and lesion‐tract overlap, indicating that observed effects were not driven by tract size; (E) Convergence with voxel‐wise disconnectome mapping. Group‐level disconnectome maps were shown alongside voxel‐wise regression results relating disconnection to adjusted AMI, demonstrating consistent involvement of left medial temporal pathways. Pearson's correlation coefficient (*r*) test was used for statistical analysis (^*^: *P* < 0.05; ^**^: *P* < 0.01; ^***^: *P* < 0.001). Abbreviations: WM, white matter; AMI, auditory memory index; SAF_ITG_MTG, basal forebrain‐amygdaloid fasciculus; CGH, cingulum‐hippocampal tract; UNC, uncinate fasciculus; ILF, inferior longitudinal fasciculus; CC_LG, corpus callosum lingual gyrus fibers; CC_MOG, middle occipital gyrus portion of the corpus callosum; MNI, Montreal Neurological Institute; ageOnset, age at seizure onset; epiDura, epilepsy duration; ASMBurden, anti‐seizure medication burden; SEEG, stereoelectroencephalography; time FU, time of follow‐up.

Disconnectome‐based analyses provided convergent evidence. Overlap of individual disconnectome maps indicated that lesions most frequently disrupted fibers coursing through medial temporal and cingulate regions. Voxel‐wise regression of individual disconnectome maps identified a focal cluster along the left CGH in which higher disconnection probability was selectively associated with poorer auditory memory performance (*z* = −2.78, *P*_FDR_ < 0.05, MNI coordinates: x = −32, y = −23, z = −8; Figure 3E).

Together, these tract‐level findings indicate that auditory memory impairment in LEAT/CCM subgroup is specifically linked to disruption of the left CGH pathway, rather than to nonspecific damage across ventral temporal white matter bundles.

### 3.4 Memory Impairment Reflected a Distributed Metabolic Network Centered on the Limbic System

Voxel‐wise NSM analyses uncovered a convergent pattern of metabolic abnormalities linked to each of the six WMS indices, and these abnormalities carried significant predictive value for individual memory function (Figure 4A). Despite interindividual variability in the spatial extent of hypometabolism, significant clusters consistently localized to the core hubs of the limbic and default mode networks, such as medial temporal, retrosplenial, and posterior cingulate cortices. These associations survived stringent voxel‐wise FDR correction, indicating that memory dysfunction in TLE reflects distributed network‐level involvement rather than isolated regional deficits (Figure 4B).

**FIGURE 4:**
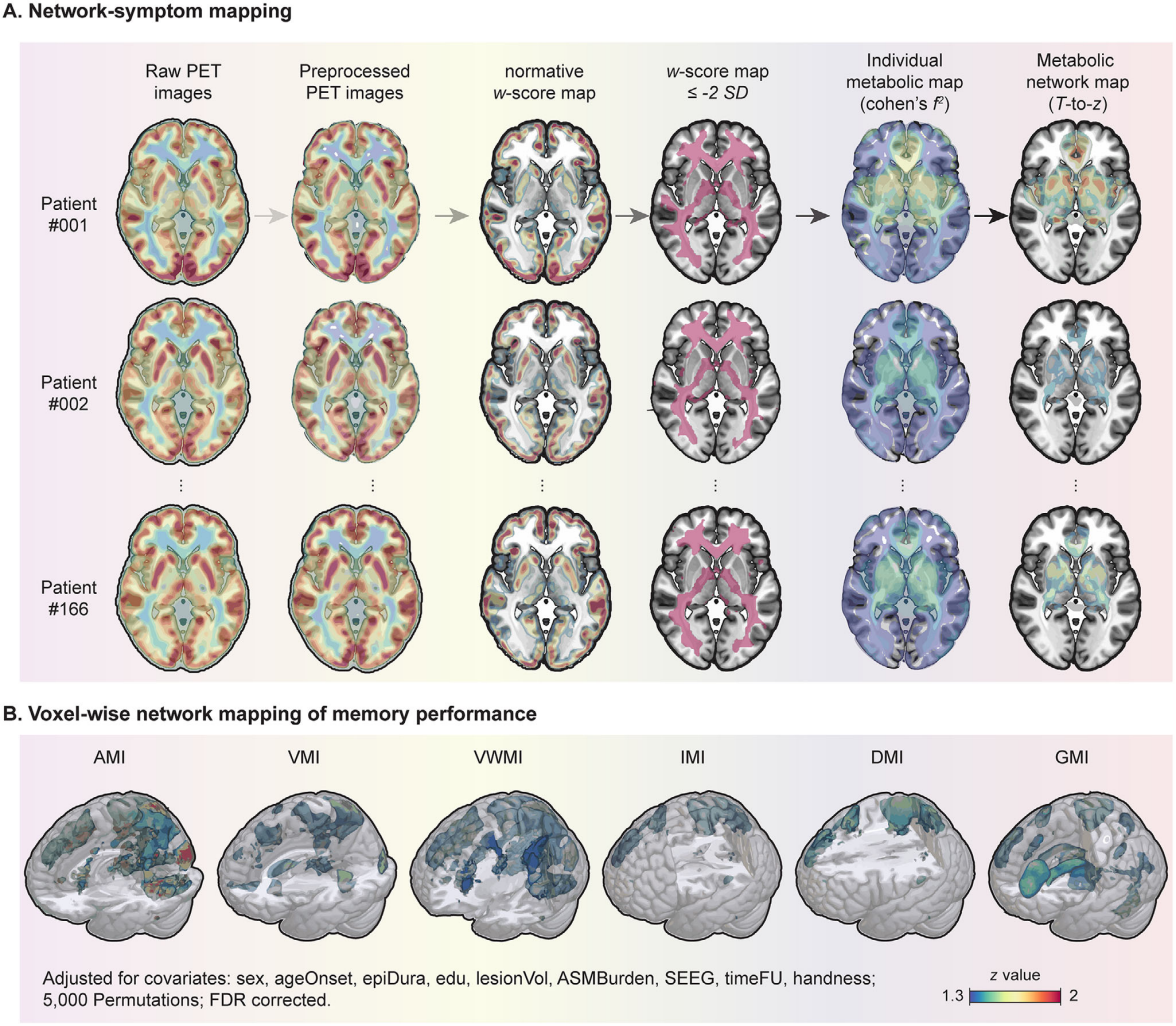
Network‐symptom mapping (NSM) analyses linking metabolic networks to memory performance. (A) Workflow of NSM analyses. A voxel‐wise general linear model (GLM) of metabolic data from controls was used to build an age‐ and sex‐adjusted normative model, and each patient's individual metabolic map was then compared with this model to generate a *w*‐map (metabolic network map; *z*‐scores adjusted for age and sex) indexing regional hypometabolism. The resulting maps were then related to memory indices and further examined with normative modelling to quantify network‐wise deviations from controls and their association with memory performance; (B) Statistical non‐parametric mapping results showing voxel‐wise regressions between metabolic network maps and each memory index in the hippocampal sclerosis/malformations of cortical development (HS/MCD) subgroup (*P*_FDR_ < 0.05, 5,000 permutations; adjusted for nuisance variables). Abbreviations: GMI, general memory index; AMI, auditory memory index; VMI, visual memory index; VWMI, visual working memory index; IMI, immediate memory index; DMI, delayed memory index; CON, control network; DAN, dorsal attention network; DMN, default mode network; LIM, limbic network; VAN, ventral attention network; SMN, somatomotor network; VIS, visual network.

Projecting FDR‐corrected NSM maps onto the Yeo 7 functional networks revealed a marked concentration of metabolic abnormalities within the limbic network (LIM) across memory domains, followed by the default mode and ventral attention networks, whereas the visual and somatomotor networks showed minimal involvement. For GMI, metabolic abnormalities were most strongly localized to the LIM, consistent with the lesion‐ and tract‐level vulnerability of the cingulum‐hippocampal pathway (Figure 5A). Sensitivity analyses showed that NSM results were highly consistent across pathological subtypes and across different thresholds used to define hypometabolism (Figure S4).

**FIGURE 5:**
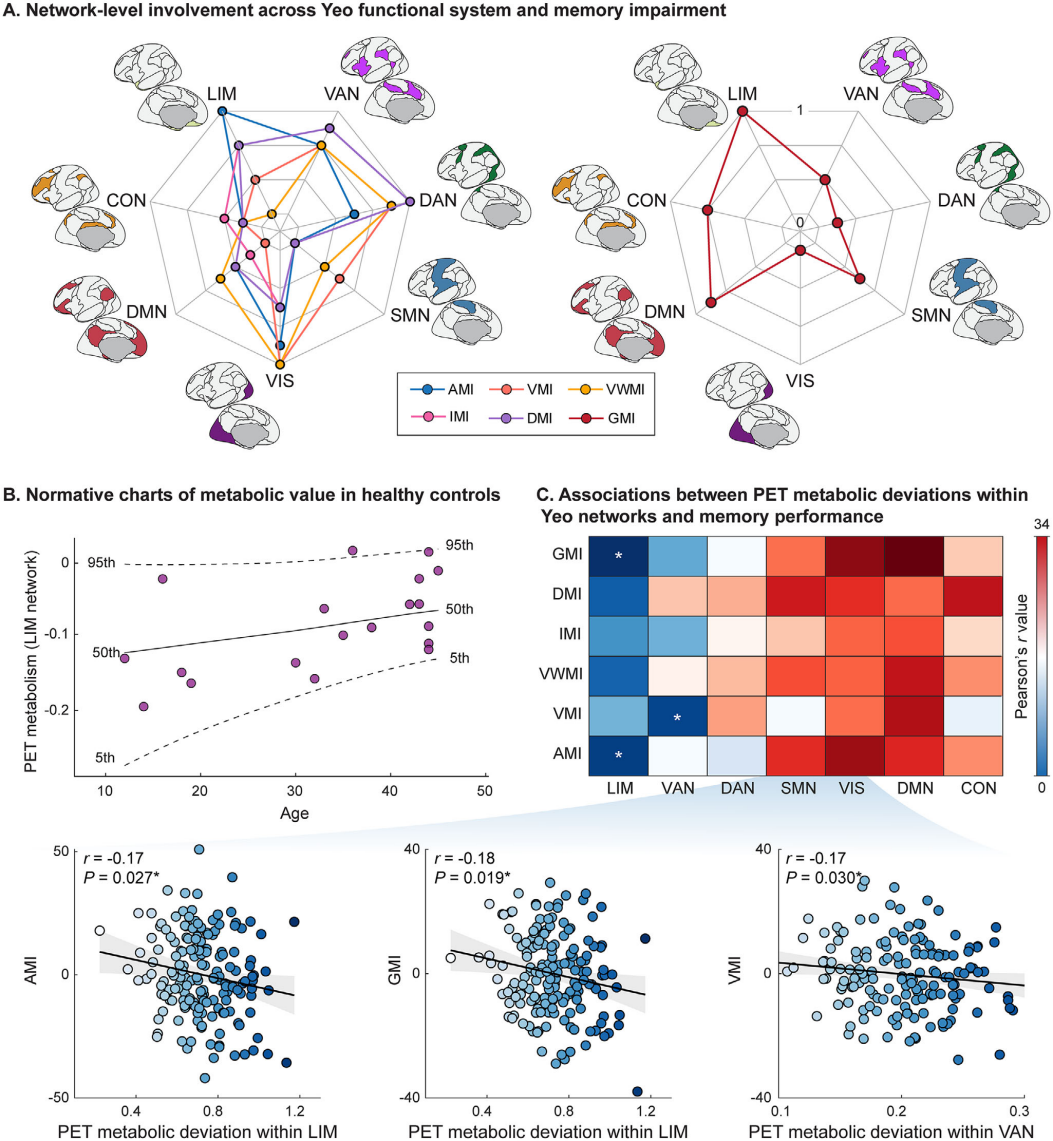
Network‐level metabolic deviations and their associations with memory performance. (A) Network‐level involvement across Yeo functional systems and memory domains. Radar plots depicted the proportion of voxels showing significant NSM effects (FDR‐corrected) within each network, representing metabolic deviation burden. The left panel showed patterns across AMI, VMI, VWMI, IMI, and DMI, while the right panel highlights the distribution for GMI. Network locations were illustrated on cortical surface maps; (B) Normative charts of PET metabolism within the LIM in healthy controls (HCs). The upper scatter showed the age‐related normative model with mean regression line and 95% prediction intervals; (C) Associations between PET metabolic deviations within Yeo networks and memory performance. The heatmap summarized the correlations between network‐level metabolic deviation scores and WMS indices, with asterisks indicating statistical significance. Scatter plots illustrated representative associations between network‐level metabolic deviations and memory performance, with linear fits and 95% confidence intervals. Abbreviations: NSM, network symptom mapping; FDR, false discovery rate; GMI, general memory index; AMI, auditory memory index; VMI, visual memory index; VWMI, visual working memory index; IMI, immediate memory index; DMI, delayed memory index; CON, control network; DAN, dorsal attention network; DMN, default mode network; LIM, limbic network; VAN, ventral attention network; SMN, somatomotor network; VIS, visual network; HCs, healthy controls.

To contextualize these network‐level effects at the individual level, age‐related normative trajectories of limbic PET metabolism were established in healthy controls using a Bayesian linear regression model with the fifth, 50th, and 95th percentile curves estimated across age (Figure 5B). Comparable age‐related normative trajectories for the remaining Yeo networks are provided in Figure S5. These normative trajectories served as the foundation for estimating patient‐level deviations. Among the Yeo networks, deviations within the LIM showed significantly negative associations with both AMI (*r* = −0.17, *P*_uncorrected_ = 0.027) and GMI (*r* = −0.18, *P*_uncorrected_ = 0.019); a similar association was observed between VAN deviations and VMI (*r* = −0.17, *P*_uncorrected_ = 0.030) (Figure 5C).

### 3.5 Molecular Systems Showed Spatial Correspondence With Metabolic Abnormalities

To explore potential molecular substrates underlying the spatial distribution of metabolic abnormalities, we performed spatial correlation analyses using a priori‐selected molecular templates representing neuromodulatory systems (e.g., serotonergic and cholinergic), core excitatory‐inhibitory neurotransmission (e.g., glutamatergic and GABAergic), and synaptic density. Among neurotransmitter systems, the serotonergic 5‐hydroxytryptamine (5‐HT_2A_), Gamma‐aminobutyric acid (GABA_A_), and synaptic vesicle glycoprotein 2A (SV2A) receptor maps showed strong positive spatial correlation with the metabolic deficit distribution (*ρ* = 0.38, *P*_FDR_ < 0.001; *ρ* = 0.37, *P*_FDR_ < 0.001; *ρ* = 0.47, *P*_FDR_ = 0.002). In contrast, the serotonin transporter (SERT) and vesicular acetylcholine transporter (VAChT) maps showed a significant negative spatial correlation (*ρ* = −0.28, *P*_FDR_ = 0.002; *ρ* = −0.27, *P*_FDR_ = 0.004).

At the cellular and mitochondrial levels, we further examined a set of markers capturing major neuronal classes and bioenergetic processes relevant to cortical organization and epilepsy. At the cellular level, the metabolic deficit pattern exhibited selective coupling with parvalbumin interneurons and mitochondrial markers indexing oxidative phosphorylation Complex II (*ρ* = 0.38, *P*_FDR_ < 0.001; *ρ* = 0.56, *P*_FDR_ < 0.001), followed by granule L3/L4 layers and Complex I. A significantly negative correlation was found for excitatory L2/3 neuronal populations (*ρ* = −0.44, *P*_FDR_ = 0.023), suggesting that bioenergetic vulnerability may contribute to memory‐related network dysfunction. Other cellular classes showed weaker or non‐significant associations (Figure 6).

**FIGURE 6:**
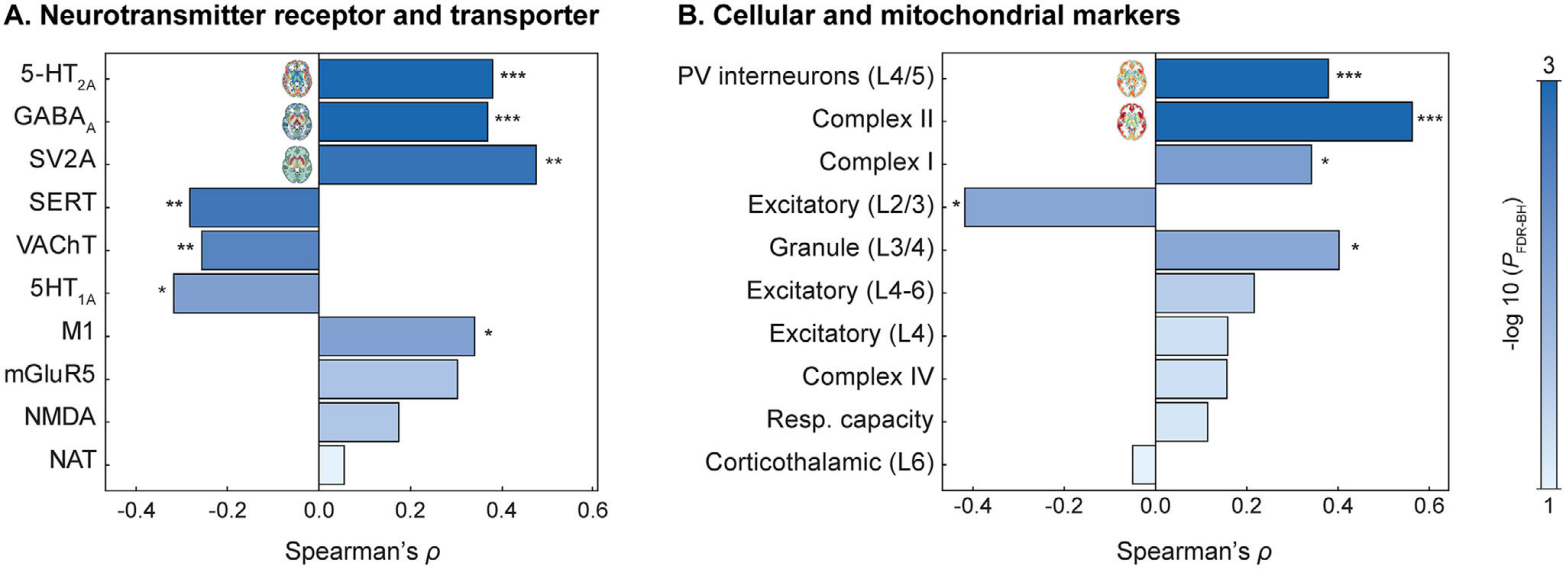
Spatial correspondence between PET metabolic abnormalities and molecular‐level markers. (A) Spatial correlations between NSM‐derived metabolic abnormality maps and neurotransmitter receptor and transporter systems. A biologically informed subset of major neuromodulatory, excitatory‐inhibitory, and synaptic markers was examined; (B) Spatial correlations with cellular and mitochondrial markers. Markers capturing major neuronal classes and bioenergetic processes were analyzed. Bars represented Spearman correlation coefficients (*ρ*), and asterisks indicate statistical significance (^*^ *P*_FDR_ < 0.05; ^**^ *P*_FDR_ < 0.01; ^***^ *P*_FDR_ < 0.001). Abbreviations: NSM, network symptom mapping; FDR, false discovery rate.

## 4 Discussion

In this study, we integrated complementary lesion‐, tract‐, network‐, and molecular‐level evidence in a large, well‐characterized cohort of LEAT/CCM and HS/MCD subgroup to delineate the multiscale substrates of memory impairment. Our findings demonstrate that memory dysfunction in TLE is closely associated with medial temporal damage and cingulum‐hippocampal disconnection, extends across a limbic‐centered metabolic network, and shows structured spatial correspondence with selected neuromodulatory, excitatory‐inhibitory, and synaptic systems, as well as mitochondrial and cellular architectures. Together, these results support a multiscale framework in which focal pathology engages distributed circuit alterations via strategic white matter pathways, disrupting limbic network organization and shaping individual vulnerability to memory impairment.

At the lesion level, our LSM results reaffirm and refine the central role of medial temporal structures in auditory memory. Auditory memory deficits in LEAT/CCM subgroup were most strongly associated with damage to the hippocampus and PHG, with a clear left‐hemispheric predominance. These regions form an integrated medial temporal system supporting auditory memory processing: the hippocampus binds multimodal sensory inputs into stable memory representations [40], and the PHG mediates the transfer of salient auditory information into medial temporal memory circuits [41]. Consistent with anatomical and functional connectivity studies, these structures are linked to the auditory association cortex via direct and indirect pathways that support sound‐evoked memory encoding and retrieval [42]. Our findings extend prior lesion‐symptom evidence of functional specialization within the temporal lobe, in which the dominant hippocampus and PHG are critical for verbal and auditory memory encoding and consolidation [6], whereas ventral temporal regions are preferentially involved in naming and semantic processing [43, 44].

At the level of WM pathways, TSM identified the left CGH as a critical conduit linking medial temporal lesions to auditory memory deficits. Although several ventral temporal and limbic association pathways, including the uncinate fasciculus and inferior longitudinal fasciculus, showed substantial lesion overlap, only the left CGH involvement was robustly and independently associated with impaired auditory memory. This finding is consistent with anatomical and functional characterizations of the cingulum bundle as a principal limbic association pathway interconnecting the hippocampus, parahippocampal cortex, posterior cingulate, and medial prefrontal regions, and supporting episodic memory integration [45]. This dissociation between lesion burden and functional impact highlights strategic white matter bottlenecks that disproportionately constrain memory performance, a concept supported by large‐scale diffusion studies in epilepsy demonstrating that tract‐specific vulnerability, rather than global white matter damage, best predicts cognitive outcome [46]. These results underscore the importance of preserving critical WM pathways during epilepsy surgery, as disruption of such tracts may not only impair memory but also precipitate downstream atrophy in connected regions [47].

Beyond focal lesions and tract disconnection, our NSM and normative modelling analyses demonstrate that memory impairment in TLE reflects dysfunction of a distributed metabolic network centered on the limbic system. Similar to recent coordinate network mapping approaches that link spatially heterogeneous abnormalities to a convergent brain network in epilepsy [48]. The limbic system is widely recognized as a core substrate for memory processes, integrating emotion, sensory context, and episodic representations across distributed circuits [49]. Consistent with prior work showing that pre‐existing brain vulnerability can interact with lesion location to shape clinical outcomes [50], our findings suggest that memory impairment in TLE reflects the interaction between intrinsic limbic network susceptibility and distributed metabolic abnormalities. In parallel, the VAN, centered on temporoparietal and ventrolateral frontal regions, has been implicated in stimulus‐driven attentional reorienting and the facilitation of visual memory processes, particularly through salience detection and externally guided encoding [51]. Although task‐based electrophysiological studies have identified temporo‐frontal regions such as the middle temporal gyrus and inferior frontal gyrus as key hubs for memory encoding, these effects are dynamic and state‐dependent [52], whereas our resting‐state metabolic analyses capture more stable, trait‐level network vulnerability, which may explain the absence of specific regional associations for IMI and DMI. Together, this supports the view that the limbic systems act as an integrated network shaping memory process, consistent with the distributed metabolic disruption observed in non‐lesional TLE.

By quantifying age‐adjusted deviations from normative network trajectories, we demonstrate that LIM deviations selectively explain interindividual variability in memory performance. This approach parallels normative modelling frameworks in psychiatric and neurodevelopmental disorders, where individual deviation maps have been used to disentangle heterogeneous patterns of brain abnormalities and link them to cognition [53, 54]. Across analytical levels, our findings indicate a multiscale circuit architecture in which focal medial temporal damage and tract‐level disconnection show convergent spatial associations with abnormal limbic metabolic organization, together delineating the systems‐level substrates of memory impairment in TLE.

By integrating spatial correlation analyses with molecular templates, we further linked these network‐level abnormalities to specific neurotransmitter and cellular architectures. Memory‐related hypometabolism showed strongest spatial correspondence with serotonergic, GABAergic, and synaptic receptor distributions. These systems converge on the regulation of synaptic plasticity, excitation‐inhibition balance, and hippocampal‐cortical interactions, and have been implicated in supporting memory encoding and retrieval processes across distributed brain networks [49]. At the cellular level, coupling with excitatory projection neuron markers and mitochondrial oxidative phosphorylation signatures suggests that memory networks in TLE may be particularly vulnerable in energetically demanding glutamatergic systems. This observation is in line with experimental and imaging studies linking mitochondrial dysfunction and excitatory neuronal stress to cognitive impairment in epilepsy and related disorders [55].

Several limitations should be acknowledged. The cross‐sectional design precludes causal inference regarding disease progression. In addition, FDG‐PET was not acquired with simultaneous EEG monitoring during tracer uptake. Therefore, subclinical seizures or interictal epileptiform activity cannot be fully excluded and may have influenced regional metabolic estimates and network‐level interpretations. Furthermore, key clinical factors known to influence epilepsy, such as cognitive decline, language lateralization, and sleep disruption, were not fully captured due to the retrospective design and may represent residual confounding. Future studies should extend this framework to longitudinal datasets, integrate SEEG‐derived network dynamics, and explore whether multiscale vulnerability markers can prospectively predict cognitive outcomes.

Together, our multiscale precision‐mapping framework reveals how temporal pathology is spatially associated with alterations across specific brain regions and WM pathways, thereby disrupting limbic‐centered metabolic networks whose vulnerability is shaped by distinct neurochemical and cellular architectures. These findings advance our understanding of memory dysfunction in epilepsy and open new avenues for personalized cognitive preservation strategies in epilepsy surgery.

## Author Contributions

Study Concept/Design: J.M.; Data Acquisition: J.M., Q.Y., and P.Z.; Writing – Original Draft: J.M. and K.Z.; Writing – Review & Editing: Q.Y., P.Z., L.S., Z.Z., B.Z., X.W., C.Z., W.H., X.S., J.Z., and Y.R.; Supervision: K.Z.

## Funding

This research was supported by grants from the National Natural Science Foundation of China (Grant No.: 82602811).

[Correction added on 1 September 2026 after first online publication: funding section is added in this version.]

## Ethics Statement

The Institutional Review Board of Beijing Tiantan Hospital (KY2020‐126‐01) approval anonymized data collection and data sharing prior to the study's commencement. All patients or their guardians gave written general informed consent for participating in scientific studies.

## Conflicts of Interest

The authors declare no conflicts of interest.

## Supporting information




**Supporting File**: advs76346‐sup‐0001‐SuppMat.docx.

## Data Availability

The data that support the findings of this study are available from the corresponding author upon reasonable request.
