# Supplementary material for: Multiscale Circuit Architecture Associated With Memory Dysfunction in Temporal Lobe Epilepsy
Source: Adv Sci (Weinh). 2026 Jul 9;13(53):e76346. doi: 10.1002/advs.76346 (PMC13348656; doi:10.1002/advs.76346)
Supplement: Supplementary file 1 [file ADVS-13-e76346-s001.docx]

**Contents**

Figure S1. Flow diagram of patient recruitment and analytic framework.

Figure S2. Associations between clinical variables and memory indices.

**Table S1.** Brain region-level proportion of significant voxels associated with auditory memory impairment

Figure S3. Associations between lesion-tract overlap and auditory memory performance across major white-matter pathways.

**Figure S4.** Sensitivity and heterogeneity analyses of network-symptom mapping (NSM) results.

Figure S5. Normative age-related trajectories of PET metabolism across Yeo functional networks in healthy controls.

Figure S1. Flow diagram of patient recruitment and analytic framework. A total of 581 patients with temporal lobe epilepsy (TLE) evaluated for surgery were screened. After exclusion based on predefined clinical and imaging criteria, 84 patients with low-grade epilepsy-associated tumor/cerebral cavernous malformation (LEAT/CCM) and 166 with hippocampal sclerosis/malformations of cortical development (HS/MCD); were included. In parallel, 52 healthy controls were recruited, of whom 33 were matched to the HS/MCD subgroup. Different analytic pipelines were applied across lesion-symptom mapping (LSM), tract-symptom mapping (TSM) and network-symptom mapping (NSM) frameworks.

Figure S2. Associations between clinical variables and memory indices. Each column shows regression lines for all patients (left), LEAT/CCM (middle), and HS/MCD subgroup (right). Each row depicts the relationship between a clinical variable and Wechsler Memory Scale (WMS) indices: age at seizure onset (top row), epilepsy duration (middle row), and years of education (bottom row). Colored lines indicate fitted linear relationships between clinical variables and WMS-IV index scores. Individual data points are omitted for clarity.

*Abbreviations*: LEAT, low-grade epilepsy-associated tumor; CCM, cerebral cavernous malformation; HS, hippocampal sclerosis; MCD, malformations of cortical development; TLE, temporal lobe epilepsy; AMI, auditory memory index; VMI, visual memory index; VWMI, visual working memory index; IMI, immediate memory index; DMI, delayed memory index; GMI, general memory index. NA, not available.

# Table S1. Brain region-level proportion of significant voxels associated with auditory memory impairment.

| **side** | **Support vector regression** | | **nonparametric Brunner-Munzel test** | |
| --- | --- | --- | --- | --- |
|  | **Brain regions** | **Proportion** | **Brain regions** | **Proportion** |
| **Left** | Hippo | 2.62% | Hippo | 2.23% |
|  | PHG | 2.17% | PHG | 1.24% |
|  | TP | 1.40% | TP | 1.26% |
|  | Amyg | 0.17% | Amyg | 1.04% |
|  | ITG | 0.93% | ITG | 0.87% |
|  | FFG | 0.32% | FFG | 0.19% |
|  | MTG | 0.02% | MTG | 0.03% |
| **Right** | PHG | 1.67% | PHG | 2.72% |
|  | FFG | 1.33% | FFG | 2.59% |
|  | Hippo | 0.11% | Hippo | 1.67% |

*Abbreviations:* PHG, parahippocampal gyrus; Amyg, amygdala; TP, temporal pole; Hippo, hippocampus; MTG, middle temporal gyrus; FFG, fusiform gyrus; ITG, inferior temporal gyrus.

Figure S3. Associations between lesion-tract overlap and auditory memory performance across major white-matter pathways. Scatter plots show Pearson correlations between the proportion of lesion-tract overlap and adjusted auditory memory index (AMI) scores for bilateral cingulum-hippocampal tract (CGH), uncinate fasciculus (UNC), inferior longitudinal fasciculus (ILF), and the ventral temporal association pathway (SAF_ITG_MTG). Shaded bands denote 95% confidence intervals. Asterisks denote statistical significance after Bonferroni correction.

**Figure S4. Sensitivity and heterogeneity analyses of network-symptom mapping (NSM) results.** (A) NSM maps stratified by pathological subtype, including hippocampal sclerosis, focal cortical dysplasia, and gliosis, showing highly consistent spatial patterns across subgroups; (B) NSM maps stratified by clinical outcome, including seizure freedom, non-seizure freedom, and loss to follow-up, demonstrating consistent network distributions across prognosis-defined subgroups; (C) NSM maps generated using different thresholds to define hypometabolism (*w*-score ≤ 1.5, -2.0 and -2.5 standard deviations), demonstrating largely overlapping network distributions across thresholds, with spatial correlation coefficients indicated.

Figure S5. Normative age-related trajectories of PET metabolism across Yeo functional networks in healthy controls. Scatter plots show PET metabolic values across age for healthy controls in six Yeo networks (VIS, SMN, DAN, VAN, CON, DMN) outside the limbic system (LIM). For each network, age-related normative trajectories were estimated using Bayesian linear regression with B-spline basis functions, yielding smooth percentile curves (5th, 50th, and 95th percentiles) that characterize the normative distribution of metabolic values across age span. These curves serve as reference distributions for quantifying patient-level deviations in the main analysis.

*Abbreviations*: CON, control network; DAN, dorsal attention network; DMN, default mode network; LIM, limbic network; VAN, ventral attention network; SMN, somatomotor network; VIS, visual network.
